# A Narrative Review of the Histological and Histomorphometrical Evaluation of the Peri-Implant Bone in Loaded and Unloaded Dental Implants. A 30-Year Experience (1988–2018)

**DOI:** 10.3390/ijerph17062088

**Published:** 2020-03-21

**Authors:** Margherita Tumedei, Adriano Piattelli, Marco Degidi, Carlo Mangano, Giovanna Iezzi

**Affiliations:** 1 Department of Medical, Oral and Biotechnological Sciences, University “G. D’Annunzio” of Chieti-Pescara, 66100 Chieti, Italy; apiattelli@unich.it (A.P.); gio.iezzi@unich.it (G.I.); 2 Biomaterials Engineering, Catholic University of San Antonio de Murcia (UCAM), Av. de los Jerónimos, Guadalupe, 135 30107 Murcia, Spain; 3 Fondazione Villaserena per la Ricerca, 65121 Città Sant’Angelo (Pescara), Italy; 4 Private Practice, 40121 Bologna, Italy; info@degidi.it; 5 Private Practice, 22015 Gravedona (Como), Italy; camangan@gmail.com

**Keywords:** dental implant, loading, osseointegration, peri-implant bone

## Abstract

*Background*: The aim of the present review was to assess the histological and histomorphometrical data from the paper published by our Laboratory on peri-implant bone in dental implants in different loading conditions. *Methods*: The papers published in different implant loading conditions, in dental implants retrieved from humans, and in the Hard Tissues Research Center of the University of Chieti-Pescara, Italy, were screened on MEDLINE/PubMed, Embase, Scopus, and other electronic databases until 31 December 2018. Only articles that reported the histological and histomorphometrical values of the Bone-Implant Contact (BIC) were selected. *Results*: The system selection provided a total of 155 papers. The manuscripts included for the narrative review were 57. These papers provided histological and histomorphometrical data. *Conclusions*: The bone remodeling around dental implants was found to be a dynamic process; loading changed the microstructure of the peri-implant bone; and implants were found to provide a successful function, over several decades, with different range of degrees of BIC in vivo (varying from a little more than 30% to a little more than 90%). Loaded implants presented a 10%–12% higher BIC values when compared to submerged, unloaded implants, and rougher surfaces had, on average, about a 10% higher BIC than machined surfaces.

## 1. Introduction

A Retrieval Bank for Implants and Biomaterials has been active since the late 80s in the Hard Tissues Research Center of the University of Chieti-Pescara in Italy. Specimens of human retrieved implants, due to different causes, have been continuously received over the last three decades, numbering now several thousands. A retrieval bank has, probably, a great scientific and clinical relevance for researchers, clinicians, and patients. The histological and histomorphometrical evaluation of such a large number of specimens could help in answering some very important questions, i.e., understanding the causative mechanisms of failing and failed implants, the relationship between implant loading and the presence or absence of mineralized tissues at the interface, and the minimum amount of bone-to-implant contact necessary to get the clinical result of osseointegration.

Bone remodeling is a prerequisite for osseointegrated dental implants to support functional loading in the long term [1,2,3,4,5,6,7,8,9,10]. Moreover, the functional loading of dental implant represents a key factor for bone development and maturation [11,12]. The continuous modification of the bone tissue around dental implant under loading results in a sequela of physiological and dynamic adaptations that involve the regional anatomy at macro- and microscopic levels [13,14,15], influencing also the response of the marginal peri-implant tissues components and collagen fibers orientation [16,17,18].

The histological characterization of osseointegration is important for a deeper understanding of bone bonding to the implant surface [19] and their capability to maintain the crestal bone level [20]. On the contrary, osseointegration can be determined only with histology, showing a bone to implant intimate contact [12]. Moreover, the evaluation, in humans, of the in vivo short- and long-term behavior of the bone tissue around dental implants in loaded and unloaded conditions could represent a substantial help in improving the long term prognosis of dental implants [11]. The value of bone-to-implant contact (BIC) plays a pivotal role in long-standing implant survival and success [7]. In a systematic review of the literature pertaining to the amount of BIC reported in retrieved dental human implants, it was found that conventionally loaded implants had a higher BIC than unloaded and immediately loaded implants, that the BIC of implants retrieved from the mandible was about 25% higher than those removed from the maxilla, that the BIC was 10% higher in the anterior when compared with the posterior mandible, and that the BIC was 25%–30% approximately higher in the anterior maxilla [7]. The received retrieved implant specimens were removed for different causes: implant fractures, psychological reasons, change of prosthetic restauration, autopsy cases, hygienic problems, and prosthetic failures. Other human samples were part of larger histological studies. The possibility to evaluate human specimens with an intact bone-implant interface could help in better understanding, e.g., of the relationship between immediate loading and the peri-implant bone response.

The aim of this narrative review was to evaluate the effect of different loading conditions, their histological/histomorphometrical evidences, and BIC findings on retrieved implants in partial or complete oral rehabilitation procedures over a 3-decade time period. The primary outcome was the percentage of bone-implant contact (BIC). A comparison was done about the different BIC values of retrieved implants.

## 2. Materials and Methods

A retrospective evaluation of all publications produced in our Laboratory in the last 3 decades was performed (M.T.), where the papers dealing with the loading of retrieved human implants were selected for the present narrative review.

### 2.1. Search Strategy

The listed PICO question, that stay for Problem/Patient/Population, Intervention /Indicator, Comparison, Outcome, was used in the present systematic search strategy: “In histological human studies, what was the outcome of retrieved loaded implants compared to unloaded retrieved implants?”.

A narrative literature review was performed on MEDLINE/PubMed, Embase, Scopus, and electronic databases through the keywords “Piattelli A AND Dental Implant AND Loading” as the search paradigm with no restrictions regarding publication date. The end of the search date was 31 December 2018. The study pool was then restricted only to histological studies in which BIC values were reported.

### 2.2. Inclusion Criteria 

In vivo studies assessing the outcome of histological evaluation of human dental implant loading were included. On the contrary, in vitro studies, animal studies, reviews, and studies with only clinical and radiological evaluation were excluded. Moreover, no limitations about the type of prosthetic connection were applied to the search.

### 2.3. Selection of the Studies 

The search data and papers selection were performed independently by two expert reviewers (M.T. and G.I.) providing a special designed electronic data form, which ensured systematic recording of data. For not available abstracts, the paper full text was obtained and evaluated. The publications not conforming to the study selection criteria were excluded. Moreover, the full text of all eligible articles was obtained and checked for inclusion criteria. For the excluded research articles, a report was performed about the reasons for exclusion.

### 2.4. Data Extraction 

The study data from included articles were extracted, collected, and evaluated. The statistical analysis was not performed due to the methodological variabilities and the different intervention sites of the studies included into the investigation.

## 3. Results

**Figure 1:**
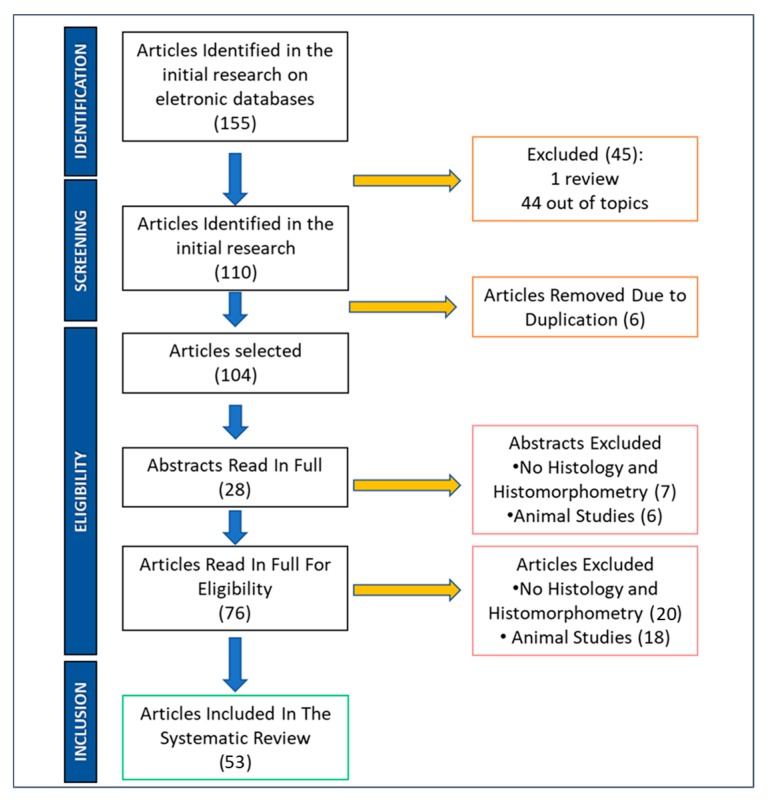
PRISMA Flow chart diagram of the study design and manuscript-selection process.

The electronic search procedure is presented in Figure 1, where the database analyses generated a total of 155 manuscripts. In this way, a title and abstract evaluation was performed in order to restrict the research field. In total, 45 articles were excluded: 1 was a review, 44 were non-pertinent to the topic, and 6 papers were removed due to duplication.

Moreover, a total of 23 papers were excluded because no histological and histomorphometrical analysis were performed, and a total of 24 papers were excluded because they were a nonhuman study.

At the end of the procedure, 53 articles satisfied the inclusion criteria and were included in this narrative review analysis for a total of 482 implants and 420 patients (Table 1). A total of 402 loaded and 80 unloaded implants were evaluated in the present review for a total of 1099 months of permanence in the oral cavity (mean: 2.28 months per implant) and a total of 982 months of cumulative loading period (mean: 2.03 months per implant).

The main characteristics of the loading protocol, implant characteristics, study outcome, and follow-up time have been summarized in Table 1. The time of service of the evaluated implants was between 4 weeks and 30 years, with a mean of 7–8 years. The reasons for retrieval were abutment fracture and other prosthetic problems in about 65% of the samples; implant fracture in about 25%; and change of prosthetic programs, hygienic problems, esthetic and orthodontic reasons, an psychological causes in about the remaining 10%. The retrieved implants were supporting a bridge in about 80% of the cases, while single implants supporting single crowns accounted for about 20% of the specimens.

**Figure 2:**
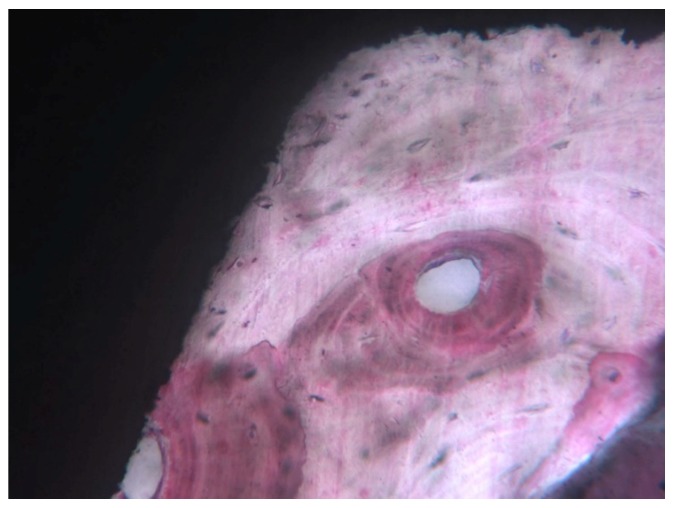
At high magnification, lamellar bone with Haversian canals were present inside the threads, in close proximity to the interface. Implant retrieved for fracture after 20 years of service: The implant supported a bridge. Courtesy of Dr. Carlo Mangano (toluidine blue and acid fuchsin staining, magnification 200×).

Most of the present histological studies on human specimens, retrieved for different causes, found compact, lamellar bone with many Haversian systems and osteons near the implant surface with a very high BIC (60%–90%) [22,23,24,25,26,27,28,29,30,31,32,33,34,35,36,37,39,40,41,42,43,44,48,49,51,53,54,55,57,59,60,65,66,67,68,69,70,72,73,74]. In a few implants, a lower BIC value, between 30% and 40%, was found. No differences were found in the BIC values in the implants retrieved for different causes. Mainly, loaded implants presented a 10%–12% higher BIC when compared with not loaded, submerged implants. A higher BIC, about 10%–12%, was reported for immediately loaded implants [38,45,46,65] (Figure 2).

**Figure 3:**
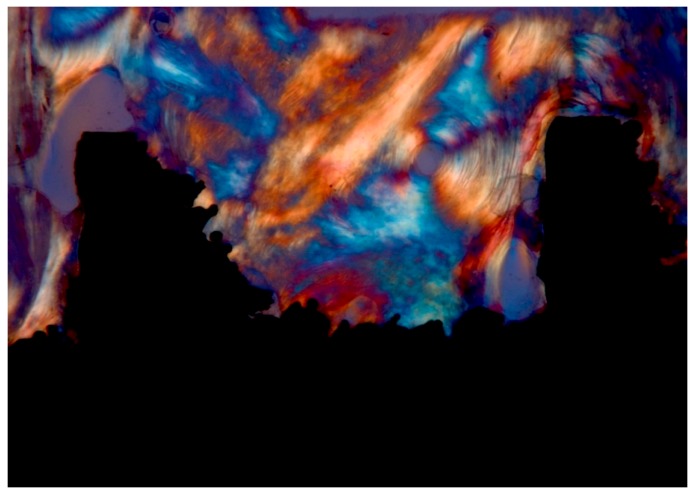
Transverse collagen fibers near an implant thread, under Compensated polarized light (CPL) microscopy: An osteon was present inside a thread. Implant retrieved for fracture after 8 years loading period: Single implant. Courtesy of Dr. Carlo Mangano. (toluidine blue and acid fuchsin staining, magnification 40×).

In loaded implants, transverse collagen fibers of the bone tissue were more abundant, while in unloaded implants, these collagen fibers of the bone tissue tended to run in a more longitudinal way (Figure 3).

**Figure 4:**
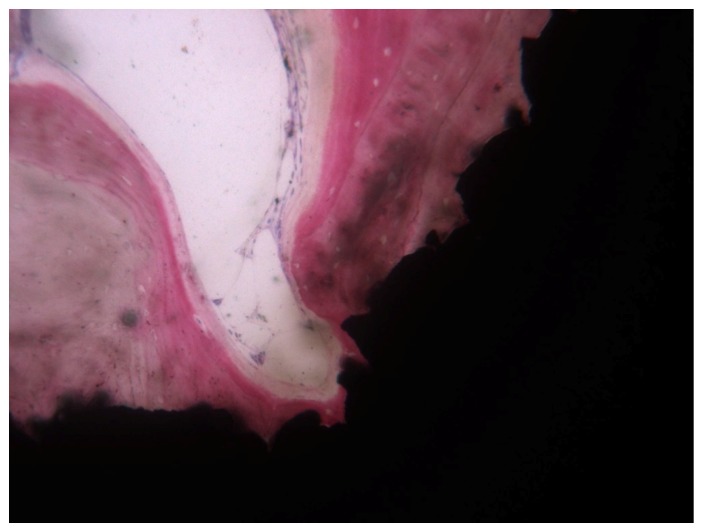
Compact, mature, and lamellar bone was in tight contact with the implant surface and adapted to all its microirregularities.Implant retrieved for esthetic reasons after 4 years loading time: The implant supported a bridge. Courtesy of Prof. Giovanna Iezzi and Prof. Adriano Piattelli (toluidine blue and acid fuchsin staining, magnification 100×).

Bone was particularly thickened around the top of the threads [73]. The main portion of these retrieved implants presented a rough surface (Figure 4), mostly sandblasted, sandblasted and acid-etched, or acid-etched surface.

**Figure 5:**
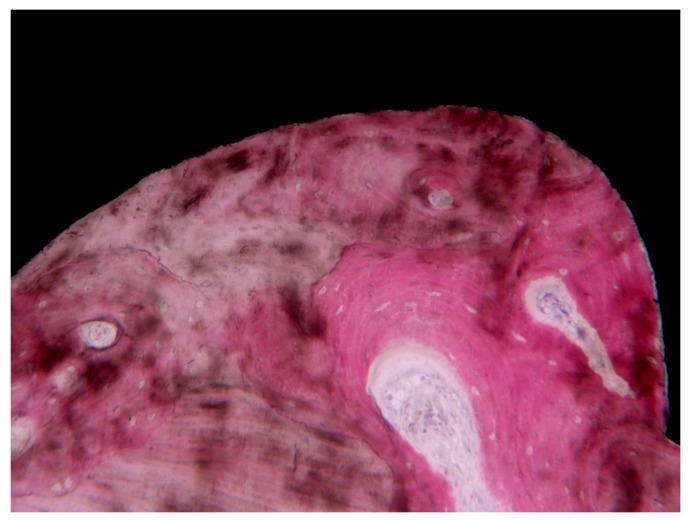
Bone remodeling areas and marrow spaces were present near the implant surface: No gaps were evident at the interface. A rim of osteoblasts making new osteoid matrix on the implant surface was evidenced. Implant was retrieved for a change in the prosthetic design after 22 years. The implant supported a bridge. Courtesy of Dr. Carlo Mangano (toluidine blue and acid fuchsin staining, magnification 100×).

Only rarely were present machined surfaces. Rougher surfaces had, on average, about a 10% higher BIC than machined surfaces. Multiple remodeled regions representing many remodeling cycles over the years were found (Figure 5).

**Figure 6:**
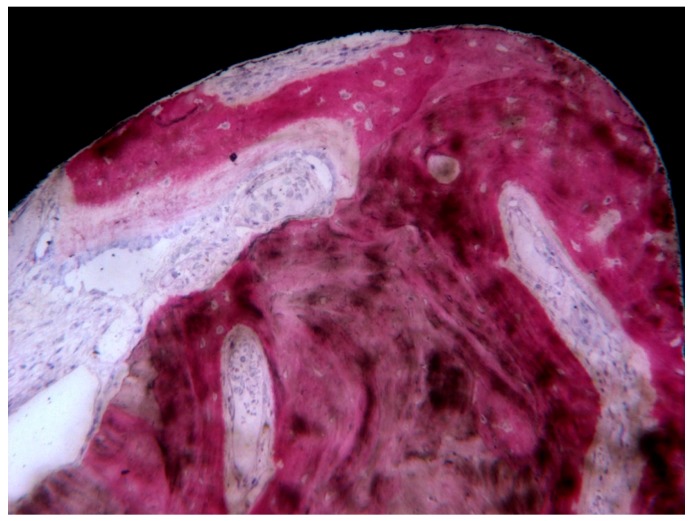
Many remodeling areas were detected. Ongoing apposition and resorption phenomena were present inside the threads. Implant was retrieved for abutment fracture after 20 years of service. The implant supported a bridge. Courtesy of Dr. Marco Degidi (toluidine blue and acid fuchsin, magnification 100×).

Bone tissue tended to adapt to loading to increase its biomechanics (Figure 6).

**Figure 7:**
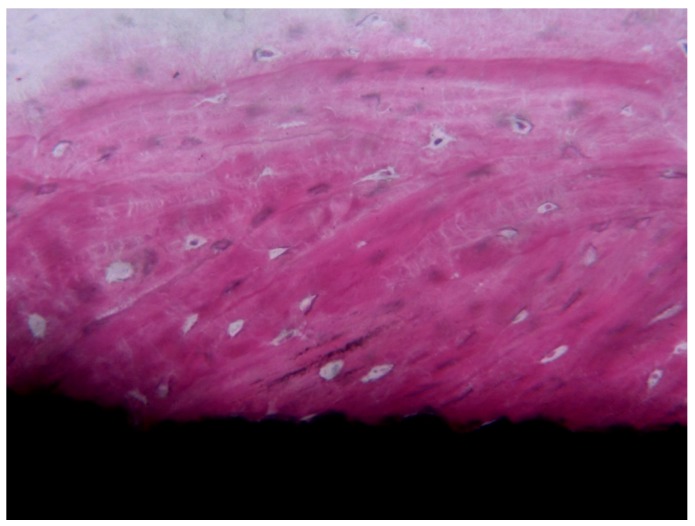
At higher magnification, many osteocytes were observed at the level of peri-implant bone, close to the implant surface. Implant was retrieved for abutment fracture after 7 years of service. The implant supported a bridge. Courtesy of Dr. Marco Degidi (toluidine blue and acid fuchsin staining, magnification 200×).

The osteocyte number was significantly higher in implants subjected to loading when compared to submerged, unloaded implants (Figure 7).

## 4. Discussion

Retrieval and histological analysis of dental implants for fracture or other reasons (such as orthodontic, psychological, esthetic, and hygienic reasons) is able to produce important data to evaluate the healing events and the interface characteristics after different time periods. The analysis of retrieved human implants is, probably, the best way to obtain an improved comprehension of the sequelae events at the level of the implant interface [68]. It is very important to document and subsequently report all these scientific observations because they play a unique role for a deeper understanding.

The reasons to assess some parameters compared to others have been introduced, tested, answered, or discussed, mainly trying to understand the causative mechanisms of failing and failed implants (the reasons for failures have been described), the relationship between implant loading, and the amount of bone at the interface (the loaded implants had a 10%–12% higher amount of BIC than unloaded implants), the minimum amount of bone-to-implant contact (BIC) needed to obtain and maintain implant stability over the long term (from a little more than 30% to a little more than 90%, and this meant that even implants with a low quantity of mineralized bone at the interface were stable and successful over the long-term), the relationship between immediate loading and peri-implant bone response (a higher BIC, 10%–12%, was found in immediately loaded implants), and the importance of the osseous tissue collagen fibers arrangement (these fibers are more able to withstand compressive loads).

Degidi et al. [65,66] reported that immediate loading did not cause untoward or adverse effects on the formation of mineralized tissues at the interface. Degidi et al. [58] reported, moreover, that loading was able to stimulate the bone remodeling at the interface; that a higher percentage of lamellar bone was found in loaded implants; that the percentage of tetracycline bone labeling was higher at the interface of loaded implants; and that a higher number of osteoblasts and osteoclasts was found in loaded implants. Traini et al. [30,56,62] reported that the implant loading seemed to determine differences in the distribution of the collagen fibers of the bone tissue in the peri-implant bone: in loaded implants, transverse collagen fibers were more abundant, while in unloaded implants, these fibers run more longitudinally. These transverse collagen fibers were mainly located at the lower flank of the threads, where compressive loads exerted their effects. Transverse collagen fibers of the bone tissue were principally located in the lower flank of the threads, where compressive loads exerted their effects.

Transverse collagen fibers have been described as the fibers most able to resist to compressive loads, and, this fact can explain their higher quantity in loaded than unloaded implants. Traini et al. [52] also found that a lower mineral density was present in the bone around unloaded implants. Guida et al. observed that immediate loading did not impair the formation of mineralized bone at the interface even in immediate post-extraction implants [50]. Barros et al. found that a higher number of osteocytes was found in the peri-implant bone around immediately loaded implants, and this fact could be related to the functional adaptation required by the loading stimulus and to the role played by osteocytes in the maintenance of the bone matrix [47]. The loading forces direction could have determined a higher mineralization of the osseous tissue located in the coronal side of the threads when compared to that in an apical location [21]. When comparing loaded and not loaded implants, different results have been provided about BIC, with higher, lower, and similar values [61,62]. In the present review, loaded implants had a 10%–12% higher BIC values when compared to submerged implants. In fact, the loading forces produced an adaptation response of the bone tissue structure [61,62]. Still unknown are the BIC values necessary for the clinical stability of an implant [3,28,57], and different values have been reported from 25% to 50% [10]. Over the years, many different types of implants have been proposed and used, such as blades, screws, and root-form implants. The screw implant has a large biomechanical retention, and a higher capability of transferring compressive forces to the peri-implant osseous tissue, producing lower shear stresses at the interface [50,65]. Macro-retention offered by the implant thread could lower the risk of implant micromovements in immediately loaded implants, improving the initial primary stability [49]. Furthermore, the threads are able to increase the surface area of the implant. A high quantity of mineralized tissue was found in an immediately loaded screw, retrieved after several years of function [48]. An osseointegrated, clinically stable, successful implant was associated with bone-to-implant contacts of at least 25% [28,29].

The limitations of the present study are well known to the authors, mainly a risk of bias due to the fact that the data came from a single institution. Most certainly, a different approach, i.e., a systematic review of the literature on this topic could have had a different scientific strength. However, it was still thought useful to conduct this study in such a way because the presented data have been accumulated over a very long time period and the histological specimens evaluated and reported were received, over the years, from several different clinicians and from several centers in different countries. Furthermore, the histological data obtained from all these specimens have shown to be very consistent. Human retrieved dental implants allowed an analysis of the short- and long-term response of bone and suffrage the effectiveness of in vitro and of animal researches. Loading of the implant changed the microstructure of the peri-implant bone, and immediate loading did not create problems to the formation of new bone at the level of implant interface and provided, probably, a positive effect on the peri-implant tissues.

Osseointegration represents a highly dynamic physiological process, and the peri-implant bone tissue increased its structure and organization over the years; these increased levels of organization are reflected by multiple remodeled areas of the bone, representing the numerous remodeling cycles over the years of functional loading. Remodeling, very well-organized, mineralized, lamellar bone was found at the interface of retrieved implants even after three decades of loading.

In all well-integrated retrieved implants, excellent bone to implant contact was found, with mineralized, mature, lamellar bone in close and tight contact with the implant surface at all regions of the implant perimeter with no sign of migration of the epithelium or formation of connective tissue. In the specimens evaluated in the present review, the bone-implant-contact percentage varied greatly from 32%–37% to more than 90%–95%. These values are quite similar to those reported by Coelho et al. [3] and Coelho et al. [11], who reported, respectively, percentages varying from about 20% to 84% (with a mean of 62%) and of 35%–95% (mean 65%). This fact meant that implants might have a successful function over a wide range of degrees of osseointegration. Moreover, implants characterized by a low bone-to-implant contact quantity were clinically stable, well-integrated, and able to provide loading conditions and functions over the years.

## 5. Conclusions

In conclusion, mineralized bone was not found in unloaded implants at the base of the threads and, in loaded implants, at their tip. Osteocytes (the mechanosensors in bone) were found in peri-implant bone, with a significantly higher number in implants subjected to loading when compared to submerged implants. Moreover, the number and thickness of bone trabeculae were significantly higher in loaded implants. In the present review, implants were shown to be stable and successful over a wide range of degrees of osseointegration (from 30% to 90%); loaded implants had a 10–12% higher BIC values when compared to submerged implants; and finally, rougher surfaces had, on average, about a 10% higher BIC than machined surfaces.

## Figures and Tables

**Table 1:** Summary of the research studies characteristics evaluated in the investigation: The implant loading protocol and the total loading time were presented in the table.

Research Studies	Results	Platform	Loading	*N*	Test	Control	Loading Time
Gandolfi et al. Int J Periodontics Restorative Dent 2018 [21]	Higher degree of mineralization of 2-month immediately loaded implant then 2-month submerged one	Screwed	Functionally loaded implants	9 implants	Functionally Loaded	Unloaded Implants	2 months to 17 years
Botticelli et al. Int J Periodontics Restorative Dent. 2019 [22]	The BIC levels of the evaluated fixtures were 83%, 66%, 74%, and 65%.	Screwed	Functionally loaded implants	4 implants	Functionally Loaded	________	14 and 17 years
Yonezawa et al. Int J Oral Maxillofac Implants 2018 [23]	BIC%: 86.8% ± 6.5% loaded. 84.6% ± 3.7% unloaded New Bone (NB): 85.5% ± 6.7% loaded; 83.4% ± 3.9% unloaded. Bone density (BD) 76.8% ± 8.3% loaded, 74.1% ± 10.5% unloaded	Screwed	Functionally loaded implants	10 implants	Functionally Loaded	Unloaded Implants	4 months
Mangano et al. Implant Dent 2017 [24]	Machined fixture: the mean BIC, bone density in the threaded area (BDTA), bone density (BD) were 21.76, 28.58, and 21.54. Dual acid-etched (DAE) fixtures were 37.49, 30.59, and 31.60.	Screwed	Functionally loaded implants	14 implants	Dual Acid-Etched (DAE)	Machined (MA) surface	2 months
Mangano C et al. Biomed res. 2017 [25]	The BIC quantity was 66.1% (±4.5%).	Screwed	Functionally loaded implants	1 patient	Metal Laser Sintered Implants	_______	5 years
Mangano F Biomed Res Int 2017 [26]	BIC% and BD%: 35.9 (±9.1) and 31.8 (±7.5). The control BIC% and BD% 29.9 (±7.6) and 32.5 (±3.9)	Screwed	Functionally loaded implants	10 patients	Anyridge®, Megagen,	EZPlus®, (Megagen)	8 weeks
Mangano et al. Clin Oral Invest 2017 [27]	A difference was reported between the two surfaces about BIC: no differences about bone density around fixtures.	Screwed	Functionally loaded implants	15 patients/ 24 implants	Nanostructured Calcium-Incorporated	Machined	8 weeks
Iezzi et al. Implant Dent. 2016 [28]	High BIC (more than 50%) was present	Screwed	Functionally loaded implants	4 implants	(A) Osseotite (3i); (B) TIXOS (Leader Italia); (C) Screw; (D) Sandblasted	_______	4/20 years function
Mangano et al. J Oral Implantol. 2015 [29]	BIC percentage varied from 37.2% to 76%	Screwed	Functionally loaded implants	4 implants	Titanium Retrieved Fixture	_______	20 years
Traini T J Biomed Mater Res B Appl Biomater. 2014 [30]	The bone-remodeling rate (BRR) was 51.9% (±10), and bone transverse collagen fibers orientation (CFO) was 13.0% (±9.7)	Screwed	Functionally loaded implants	5 implants	Implant Fractured Retreved	_______	20 years
Piattelli A J Biomed Mater Res B Appl Biomater. 2014 [31]	Osteocyte density higher at 1–5 years and decreased at 14–27 years loading	Screwed	Functionally loaded implants	18 implants	Titanium Implant	_______	4 weeks to 27 years loading
Iezzi et al. Odontology. 2014 [32]	The BIC of the three best threads for all implants varied from 94% to 100%	Cone morse	Functionally loaded implant	8 implants	Retrieved Implants	_______	8 years loading
Mangano et al. Implant Dent. 2013 [33]	Superficial debris and particle inclusions around the tissues close to the bone	Screwed	Functionally loaded implants	2 implants	Laser Metal Sintering	_______	8 weeks
Mangano et al. Int J Oral Maxillofac Implants. 2013 [34]	Compact, mature lamellar bone was present.	Screwed	Functionally loaded implants	2 implants	Sandblasted, Acid-Etched	_______	5, 10 years
Iezzi et al. Int J Periodontics Restorative Dent. 2013 [35]	An increased BIC was reported around the dental microstructured fixtures	Cone morse	Functionally loaded implants	4 implants	Titanium Fixture	_______	>1 year
Iezzi et al. Implant Dent. 2013 [36]	No epithelial ingrowth was reported. A very high BIC quantity was reported	One piece	Functionally loaded implants	14 implants	Titanium Fixture	_______	4–8 weeks
Degidi et al. Clin Implant Dent Relat Res. 2013 [37]	Variable torque work (VTW) shows a negative significant correlation with initial BIC in bone I and a positive significant correlation in bone II and III.	Screwed	Immediately loaded implant	90 implants	Porous Anodized Surface.	_______	Immediate
Shibli et al. J Periodontol. 2013 [38]	BIC levels were 45.20% ± 7.68% and 34.10% ± 7.85% for immediately loaded (IL) and unloaded (UI) implants	Screwed	Immediately loaded implants	12 patients, 24 implants	Laser Implants Immediately Loaded	Direct laser Implants	8 weeks
Mangano et al. J Periodontol. 2012 [39]	The first visible bone contact was 0.28 ± 0.30 mm (95% confidence interval, 0.24 to 0.32)	One-piece	Immediately loaded implant	96 implants	Tixos Nano Ovd	_______	1 year
Iezzi et al. Quintessence Int. 2012 [40]	The BIC of machined fixture was 92.7%. The sandblasted fixture reported a BIC of 85.9% and 76.6%.	Screwed	Functionally loaded implants	3 implants	Two-Stage Submerged Implant	_______	5 years
Degidi et al. Int J Oral Maxillofac Implants. 2010 [41]	A statistically nonsignificant correlation was present between resonance frequency analysis (RFA) values and BIC.	Screwed	Functionally loaded implants	16 implants	Sandblasted And Acid-Etched	______	4 or 8 weeks
Shibli et al. J Biomed Mater Res A. 2010 [42]	The BA% was higher for the direct laser fabrication (DFL) surface, although there was no difference with the SAE surface.	Cone morse	Functionally loaded implants	30 patients	Direct Laser Fabrication Implant	______	8 weeks
Mangano et al. J Oral Implantol. 2010 [43]	The mean of BIC percentage was 69.51%.	One piece	Unloaded implant	1 implant	Laser Sintering Procedure	_______	_________
Shibli et al. Clin Implant Dent Relat Res. 2010 [44]	Increased BIC and osteocyte index (Oi) test group and no BA% differences	Cone morse	Functionally loaded implants	20 implants	Dual Acid-Etched Surface And Bioceramic Molecular Impregnated	_______	2 months
Degidi et al. Int J Oral Maxillofac Implants. 2009 [45]	The BIC in implant A reported a value of 51.2% ± 4.5% whereas, in implant B, was 55.1% ± 2.3%	Screwed	Functionally loaded implants	2 implants	Functionally Loading Implants	Unloaded Implants	5 weeks
Degidi et al. Int J Oral Maxillofac Implants. 2009 [46]	The IL BIC was 76.2% and the submerged BIC 62.3%	Screwed	Immediately loaded implants	4 patients	Submerged Dental Implants	_______	4 and 8 weeks
Barros et al. J Periodontol. 2009 [47]	A higher quantity of osteocytes was reported around IL implants	Screwed	Immediately loaded implant	14 patients, 28 implants	Immediately Loaded	Submerged dental	8 weeks
Iezzi et al. J Osseointegr 2009 [48]	The BIC value was 75% ± 4%	One piece	Immediately loaded implant	1 implant	Immediately Loaded	_________	12 years
Vantaggiato Implant Dent. 2008 [49]	The BIC percentage was 56.3% ± 5%	Screwed	Immediately loaded implants	3 implants	Immediately Loaded Screw Implants	________	3 years
Guida et al. J Periodontol. 2008 [50]	The BICs of control and test were 58% ± 4.0% and 52% ± 3.2%	Screwed	Immediately loaded implant	1 implant	Loaded Immediately	_________	6 months
Degidi et al. Clin Oral Implants Res. 2008 [51]	The BIC levels were 65.3% ± 4.8%	Cone Morse	Immediately loaded implants	3 implants	Cone Connection	_________	4 weeks
Traini et al. J Dent. 2007 [52]	The low mineral density index (LMDI) was of 29.2 ± 3.1, while the high mineral density index (HMDI) was of 88.2 ± 3.6	Screwed	Unloaded implants	5 implants	Micro-Structured Surface Xive Implant	_________	6 months
Iezzi et al. Implant Dent. 2006 [53]	The bone showed lamellae that tended to run parallel to the implant surface	Screwed	Immediately loaded implants	3 implants	Immediately Loaded Implants	_________	4 months
Di Stefano et al. J Oral Implantol 2006 [54]	The BIC was 51% ± 6%	Screwed	Immediately loaded implant	1 implant	Immediately Loaded Blade Implant	_________	20 years
Traini Implant Dent. 2006 [55]	The BIC percentage was 81.6% ± 1.5%	Screwed	Functionally loaded implant	1 implant	1 Fractured Screw-Shaped Implant	_________	5 years
Traini et al. J Oral Implantol. 2006 [56]	The BIC of implant A was 67.9% ± 9.5% whereas, for implant B, was 74.6% ± 11.2%	Screwed	Functionally loaded implants	2 implants	1 Short-Term Implant (Implant A)	1 long-term implant (implant B)	4 months, 12 years
Romanos J Periodontol. 2005 [57]	A high BIC level of 66.8% (±8.9%) was reported	Screwed	Immediately loaded implants	29 implants	Immediately Loaded	_________	2 and 10 months.
Degidi et al. J Oral Implantol. 2005 [58]	The BIC level was 71% ± 3.2%	Screwed	Immediately loaded implant	case report	Immediately Loaded Implant	_________	2 months
Iezzi J Oral Implantol. 2005 [59]	The BIC level varied from 30% to 96%	Screwed	Unloaded	8 implants	Submerged, Unloaded Implants	_________	12 months
Degidi et al. J Long Term Eff Med Implants. 2005 [60]	BIC percentage was 72.6% (±2.7%)	Screwed	Immediately loaded implant	case report	Immediately Loaded Implant	_________	14 months
Traini et al. J Biomed Mater Res B Appl Biomater. 2005 [61]	Peri-implant bone area (PB) 32.96% (3.208 ± 0.435 mm) with transverse collagen fibers 19.70% (1.957 ± 0.253 mm) by longitudinally collagen fibers	Screwed	Immediately loaded implants	10 patients, 20 implants	Implants Retrieved	_________	6 months
Traini J Periodontol. 2005 [62]	Transverse collagen fiber 45,481 ± 3037 pixel^2^ and longitudinal collagen fibers 13,676 ± 2232	Screwed	Immediately loaded implants	10 implants	Immediately Loaded Implant	unloaded implant	6 months
Degidi et al. J Oral Implantol. 2005 [63]	Lamellar bone, osteoblasts, and bone tetracycline labeling higher loaded implants	Screwed	Immediately loaded implants	12 patients	Immediately Loaded	Unloaded implant	6 months
Degidi et al. J Oral Implantol. 2003 [64]	The BIC percentage was about 65% to 70%	Screwed	Immediately loaded implants	3 implants	Immediately Loaded	submerged	6 months
Degidi et al. Clin Implant Dent Relat Res. 2003 [65]	The BIC level was 60 to 65% for all fixtures	Screwed	Functionally loaded implants	6 patients, 11 implants	Immediately Loaded Titanium Implants	_________	10-month
Degidi et al. Clin Implant Dent Relat Res. 2003 [66]	BIC level was 80.6% ± 4.7%.	Screwed	Immediately loaded implant	2 implants	Immediately Loaded Titanium Implants	_________	6 months
Degidi Clin Implant Dent Relat Res. 2002 [67]	The BIC percentage was about 60%	Screwed	Immediately loaded implant	case report	Porous Anodized surface	_________	6 months
Testori et al. Int J Periodontics Restorative Dent. 2001 [68]	BIC ranging from 78% to 85%	Screwed	Immediately loaded implants	12 implants	Immediate Loading	Submerged	6 months
Piattelli J Periodontol. 1997 [69]	The BIC percentage of about 60%	Screwed	Functionally loaded implant	1 patient	Titanium Non-Submergedplasma-Sprayed Implants	_________	10-month
Piattelli Int J Oral Maxillofac Implants. 1997 [70]	Mature bone at the level of the surface of the implant	Screwed	Functionally loaded implant	1 patient	Retrieved Dental Implants	_________	10 years loading
Piattelli J Periodontol. 1997 [71]	The BIC percentage ranged 60 to 70%.	Screwed	Immediately loaded implants	2 patients	Titanium Plasma-Sprayed (TPS)	Machined	8 and 9 months
Piattelli Biomaterials. 1996 [72]	The fixtures were surrounded by compact and mature lamellar bone	Screwed	Early loaded implants	2 patients	Titanium Implants	_______	18 and 42 months
Piattelli J Oral Implantol. 1993 [73]	The BIC of 86.69% (SD = 5.43)	Screwed	Early loaded implant	1 patient	Early Loaded	_______	7 years
Trisi J Periodontol. 1993 [74]	Structures similar to bone reversal lines were observed at the edge of the bone side of the interface	Screwed	Functionally loaded implants	1 patient 2 implants	Blade Implant	_______	7 to 20 years

*N*: Number of Implants; BIC: bone to implant contact, NB: New Bone; BD: Bone density, BDTA: density in the threaded area, BD: bone density, BRR: bone-remodeling rate, bone CFO: transverse collagen fibers orientation, Oi: osteocyte index, VTW: Variable torque work; IL: immediately loaded, UI: unloaded implants; RFA: resonance frequency analysis, LMDI: low mineral density index, HMDI: high mineral density index, PB: Peri-implant bone area.
